# Comparison of under-mattress sensor and CPAP data for home monitoring in obstructive sleep apnea

**DOI:** 10.1007/s11325-026-03741-9

**Published:** 2026-06-22

**Authors:** R-Y Yang, T. Seailles, N. Aisenberg, O. Felix, A. Sabil, P. Escourrou

**Affiliations:** 1 Withings - Issy les Moulineaux (France), Issy-les-Moulineaux, France; 2 Centre Médical -Montesson, Montesson, France; 3 Cabinet ORL-Bry sur Marne, Bry sur Marne, France; 4 SomniPlanet - Courbevoie, Courbevoie, France; 5 Cloud Sleep Lab- Paris, Paris, France; 6 Centre Interdisciplinaire du Sommeil, 35 rue de Lubeck, Paris, 75116 France

**Keywords:** Obstructive sleep apnea, Continuous positive airway pressure therapy, Telemonitoring, Remote patient monitoring, Treatment adherence, Withings sleep analyzer

## Abstract

**Purpose:**

To evaluate sleep duration and respiratory events in comparison with CPAP use time and residual events.

**Methods:**

We evaluated 75 OSA patients using a non-intrusive under-mattress sensor (WITHINGS Sleep Analyzer, WSA) alongside CPAP Resmed Airsense device over 480 nights, with a subset of 14 patients undergoing simultaneous home polysomnography (PSG). We compared total sleep time (TST) with CPAP use time and residual apnea-hypopnea index (AHI) across devices.

**Results:**

The WSA recorded longer TST than CPAP use time (437.4 vs. 395.6 min, *p* < 0.005) and higher AHI than CPAP residual AHI (11.3 vs. 2.0 events/h, *p* < 0.001). In the PSG subset, WSA AHI closely approximated PSG AHI and exceeded CPAP residual AHI.

**Conclusions:**

The WSA provides a more comprehensive home monitoring of sleep and respiratory events during CPAP therapy, potentially improving treatment assessment and patient engagement, especially in early treatment phases.

**Supplementary Information:**

The online version contains supplementary material available at 10.1007/s11325-026-03741-9.

## Introduction

Moderate or severe obstructive sleep apnea (OSA) is very prevalent in the adult population (up to 49·7% in men) [1, 2] and is associated with serious health consequences, including hypersomnolence leading to accidents and increased risk of cardiometabolic diseases such as high blood pressure, coronary heart disease, heart arrhythmia and stroke [3]. The first-line treatment for sleep apnea is Continuous Positive Airway Pressure (CPAP) whose effectiveness depends on the type of machine and mask, the extent of leakage and patient adherence [4]. However, CPAP treatment can be cumbersome due to its interface and the noise of the machine, which may disturb the patient’s sleep. Although adherence to positive airway pressure (PAP) therapy for ≥ 4 h/night is associated with a significant reduction in the risk of secondary cardiovascular events [5, 6] fewer than 50% of patients use CPAP for > 4 h/night after 1 year [7], and up to 48% discontinue treatment at 3 years [8]. To improve adherence, telemonitoring-guided interventions using data from CPAP devices have been recommended during the initial period of therapy [9]. However these interventions are associated with a modest increase in CPAP use (~ 29 min/night) and a small increased likelihood of ≥ 4 h/night usage [10]. CPAP machines can record and remotely transmit data on usage times, mask leaks and residual respiratory events. However, these devices only record data during periods of use: if the mask is disconnected voluntarily or unintentionally during the night and the patient returns to sleep, the machine stops recording. As a result, patients may receive treatment for only a portion of their total sleep time often at the beginning of the night when the therapy is better tolerated. Further episodes of apnea can occur particularly during Rapid Eye Movement (REM) sleep which predominates towards the end of a night and is often when apneas are most severe [11]. Therefore it has been proposed that determining the overall effectiveness of CPAP, accounting for sleep time, user hours, and actual CPAP efficacy, may provide new insights into the treatment in OSA [12].

Treatment effectiveness also depends upon the residual respiratory events which are derived from the flow signal of the CPAP through proprietary automatic event detection (AED) algorithms. However, studies have shown that CPAP are still limited in terms of accuracy, especially due to the lack of standardization among detection algorithms, and there are growing concerns about relying solely on AED functions compared to actual flow curve analysis and PSG analyses [13–16]. Therefore independent measurements of residual respiratory events is desirable but impractical to monitor these on several nights by polygraphy or polysomnography. There is a need for easy, affordable and validated consumer wearables to monitor on the long term, CPAP reliability and to compare CPAP use time with actual sleep time when initiating and following the treatment in the home setting [17]. The aim of this study was to compare CPAP-derived usage and residual AHI with sleep time and event rates measured by a consumer under-mattress sensor (nearable device), and to benchmark both devices against home PSG in a subset of patients undergoing OSA treatment.

## Methods

Participants were men and women aged 18 years or older with a diagnosis OSAS, based on clinical symptoms, such as snoring, witnessed apneas, daytime fatigue or sleepiness, and an AHI ≥ 15 events/hour measured by polygraphy or polysomnography (NOX medical Reykjiavik Iceland, CIDELEC Ste Gemmes sur Loire France, using AASM 2017 criteria) [18]. Subjects with severe cardiopulmonary or neuromuscular disease were excluded. All subjects were recruited from six sleep centers in the Paris region. Written informed consent was obtained from all participants, in accordance with the ethically approved protocol (ID-RCB N°:2020-A03578-31). Two home healthcare providers (HHCP), SomniPlanet and SOS Oxygène, were responsible for the management and monitoring of patients.

### Procedures

Patient’s baseline data were collected by the investigators after consent.

The patient had to undergo CPAP treatment with a CPAP machine for OSA for at least one month. This condition ensured that the patient was compliant to its treatment (CPAP) and mask and mouth leaks were corrected by the home care provider.

The Withings sleep analyzer (WSA), is a CE medical marked and FDA cleared connected pneumatic sensor placed under the mattress at the thorax level. It records changes in pressure resulting from body movement, chest displacement during breathing, and vibrations from cardiac contractions. These signals are filtered in different frequency bands to provide data on movement, breathing and heart beats. The device also includes a microphone, to extract a snoring signal [19]. The WSA is a commercialized stand-alone device with embedded software that processes and analyzes these signals to detect sleep stages and apnea-hypopnea events. Data collected by the WSA are transmitted to the WITHINGS servers via mobile or Wi-Fi network. The device does not come into contact with the patient and require no operation after installation. The WSA-estimated AHI has shown good agreement with in-laboratory polysomnography-derived AHI, with 88% sensitivity and 88% specificity for classifying moderate-to-severe OSA [20, 21] similar to other wearables and nearables devices [22].

Two types of CPAP were planned for use during the study according to the physician responsible for the patient: RESMED Airsense S10 and PHILIPS DS1.

All CPAP and WSA data were recorded at the homes of participating patients. The technician from the HHCP, who previously had installed the CPAP machine at the patient’s home, set up the WSA during the routine check-up visit after the 1 st month of treatment and ensured that data were collected from the CPAP machine during 7 days after having installed the WSA, either by arranging another visit, or remotely if the CPAP machine’s remote transmission was functioning. Seven nights of CPAP data were extracted from the CPAP telemonitoring reports.

The data were anonymously stored and associated with a participant based on the device MAC address. The WSA used a study firmware specifically developed to work without the companion Health Mate App and therefore only transmitted unidentifiable data. The CPAP data were stored on the Health Data Servers of the CPAP manufacturers and the servers of the homecare providers. The WITHINGS Sleep data were not shared with the investigators nor with the patients during the entire inclusion period and the anonymized data from the CPAP reports were not communicated to WITHINGS during the entire inclusion period. To avoid any potential influence of the compensation provided by WITHINGS for clinicians’ time dedicated to patient recruitment and data analysis, the unblinding process was conducted only after the inclusion of the last patient.

#### Statistical analysis

**Fig. 1:**
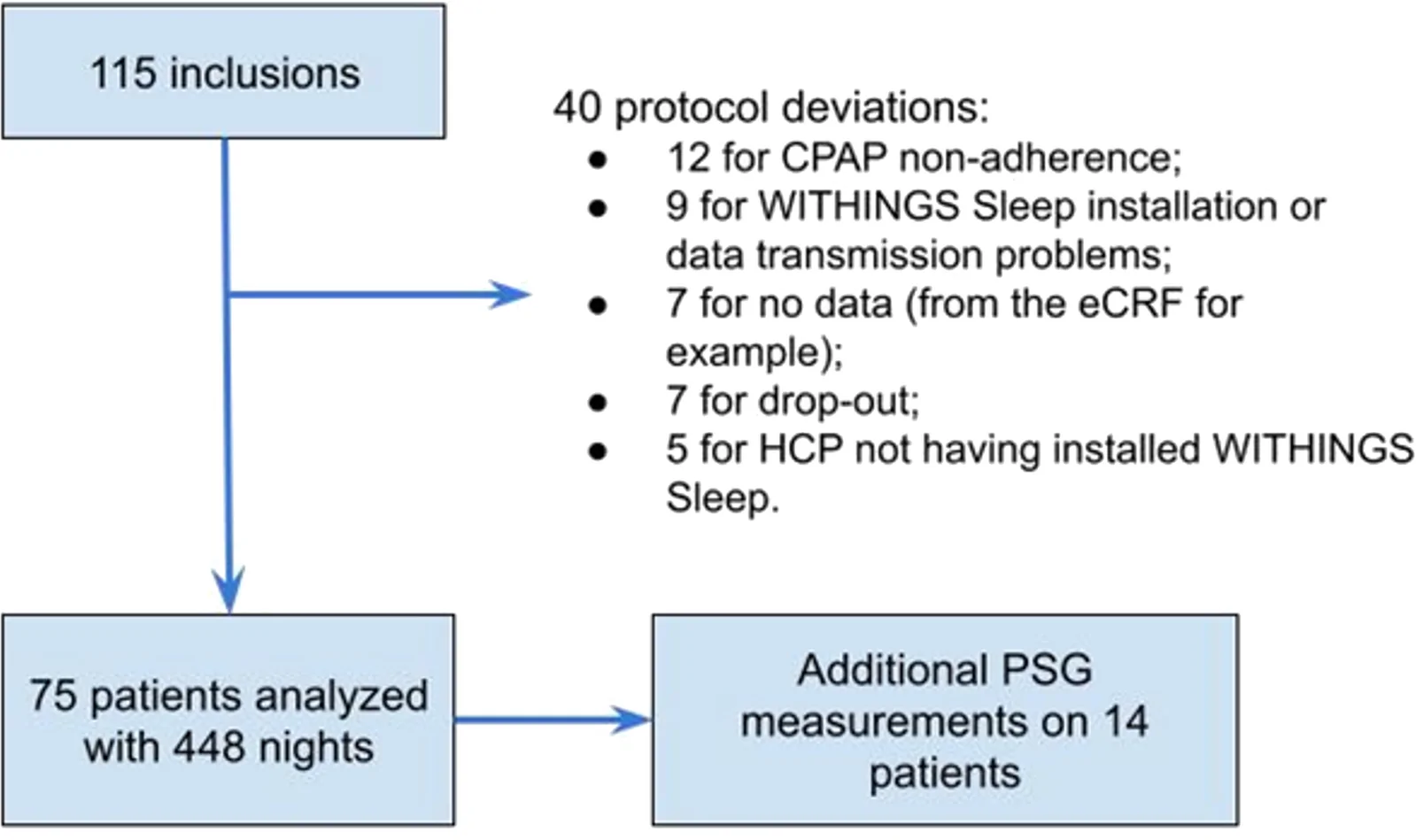
flow-chart of patients: inclusion, dropouts (reasons), N recordings CPAP/Sleep, N PSG/CPAP/Sleep

The hypothesis was an absence of difference between the AHI of the CPAP machines and the AHI estimated by the WITHINGS Sleep Analyzer. The sample size of patients to be included, for a type I error of 0.05 and a type II error of 0.10, assuming a clinically significant average of the expected differences between the machine AHI and the WITHINGS Sleep analyzer of 5/hour and an expected standard deviation of the differences between AHI of 8/hour, was 30 per machine, i.e. 60 patients with at least one night of analyzable sleep data and CPAP recordings. The statistical analyzes were carried out with the Python and R libraries after reviewing the data to identify deviations from the protocol and their potential impact on the analyses. A significance level of alpha = 0.05 was used for all the analyses. When the data was not normally distributed, the Wilcoxon test was used. To consider patients with a sleeping time of < 5 h (minimum sleeping time necessary for an AHI determination by the WSA), the sample size was increased by 25% to obtain 60 analyzable patients, i.e. 75 patients. 115 participants were included in the study. Among them there were 40 protocol deviations (Fig. 1: Patient flow chart). Missing data at the time of analysis were not replaced. In total 75 patients with 480 nights had readable/classifiable paired recordings from the WSA and CPAP. Due to the worldwide PHILIPS DS1 CPAP recall, the study used only one CPAP device: RESMED Airsense S10.

Additionally at home PSG with CPAP and WITHINGS Sleep Analyzer have been prescribed at random to patients volunteering for this procedure. The sleep technician set up the PSG after dinner and came back in the morning to disconnect the patient. Thus, an additional analysis has been performed on 14 patients from 2 sites, simultaneously with the PSG, the WSA and the CPAP recordings. The PSG were performed with a CIDELEC recorder and analysed according to AASM 2017 rules (AHI 3%) blinded to the results of CPAP and WSA.

## Results

**Fig. 2:**
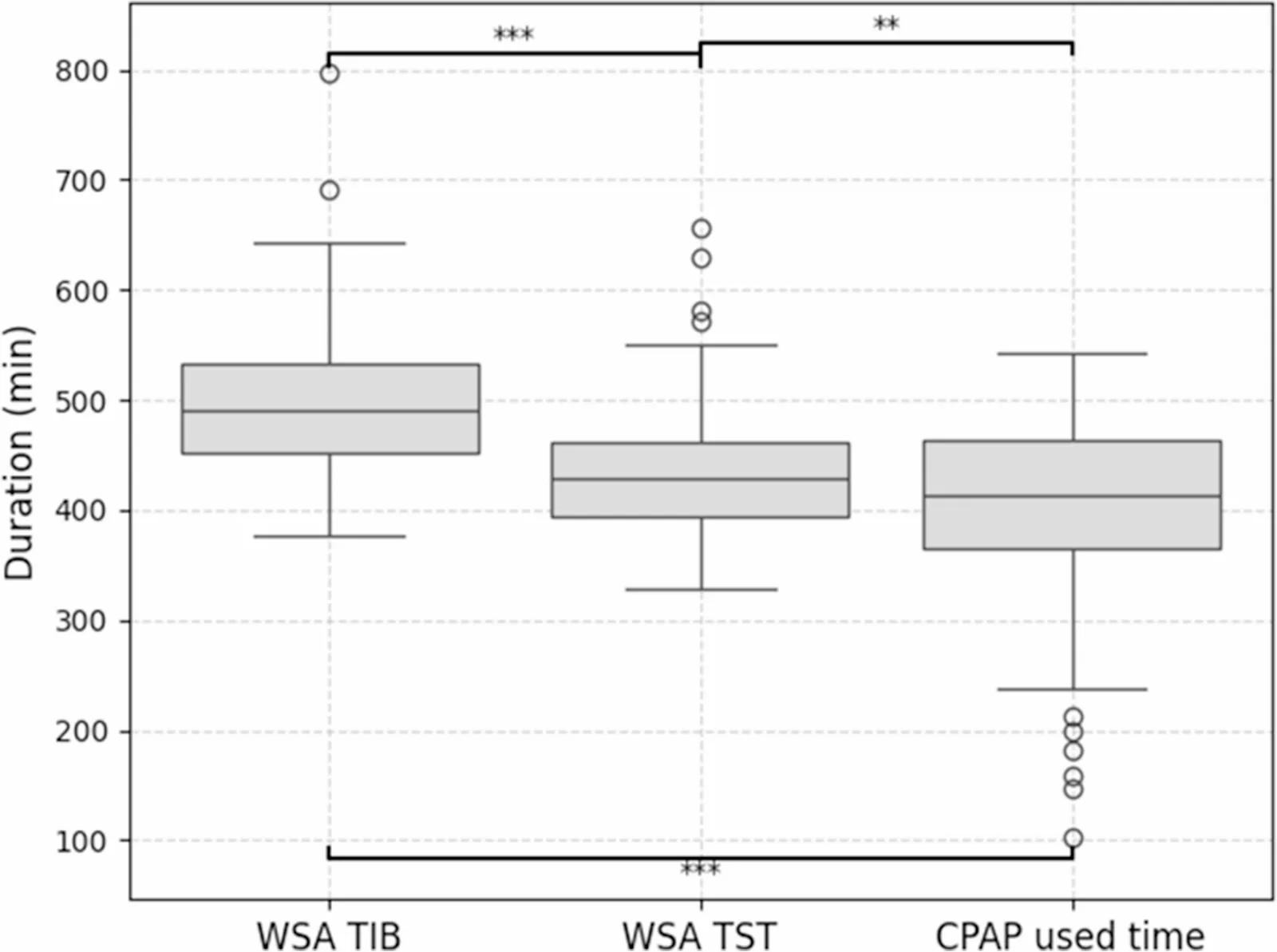
Comparison WSA Time in bed, Total Sleep Time/CPAP durations (min). Shown are box and whisker plots with the median (central line). The points at the whisker are the 2.5 and 97.5% values

**Fig. 3:**
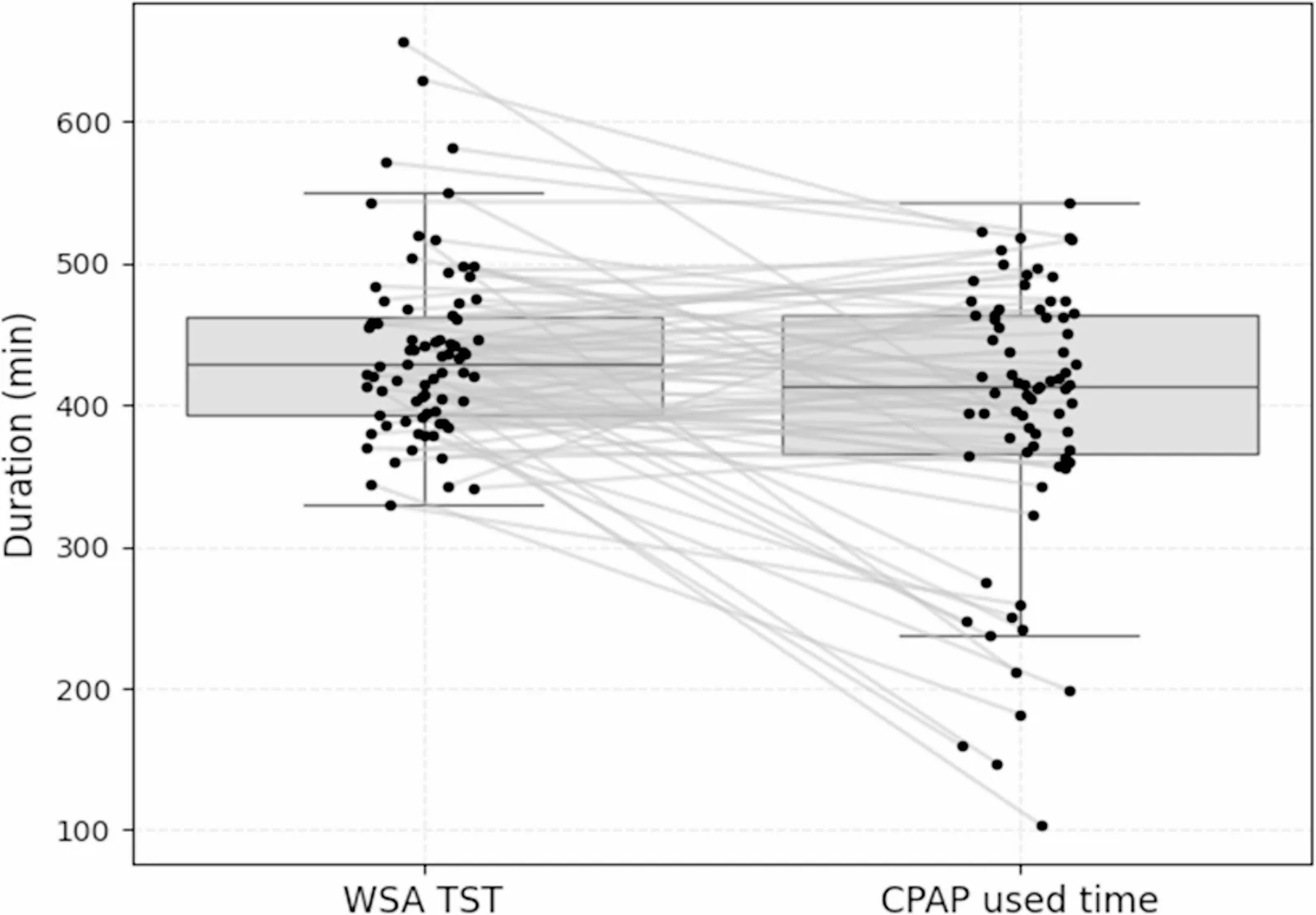
Individual comparison of WSA Total Sleep Time and CPAP durations (min)

**Fig. 4:**
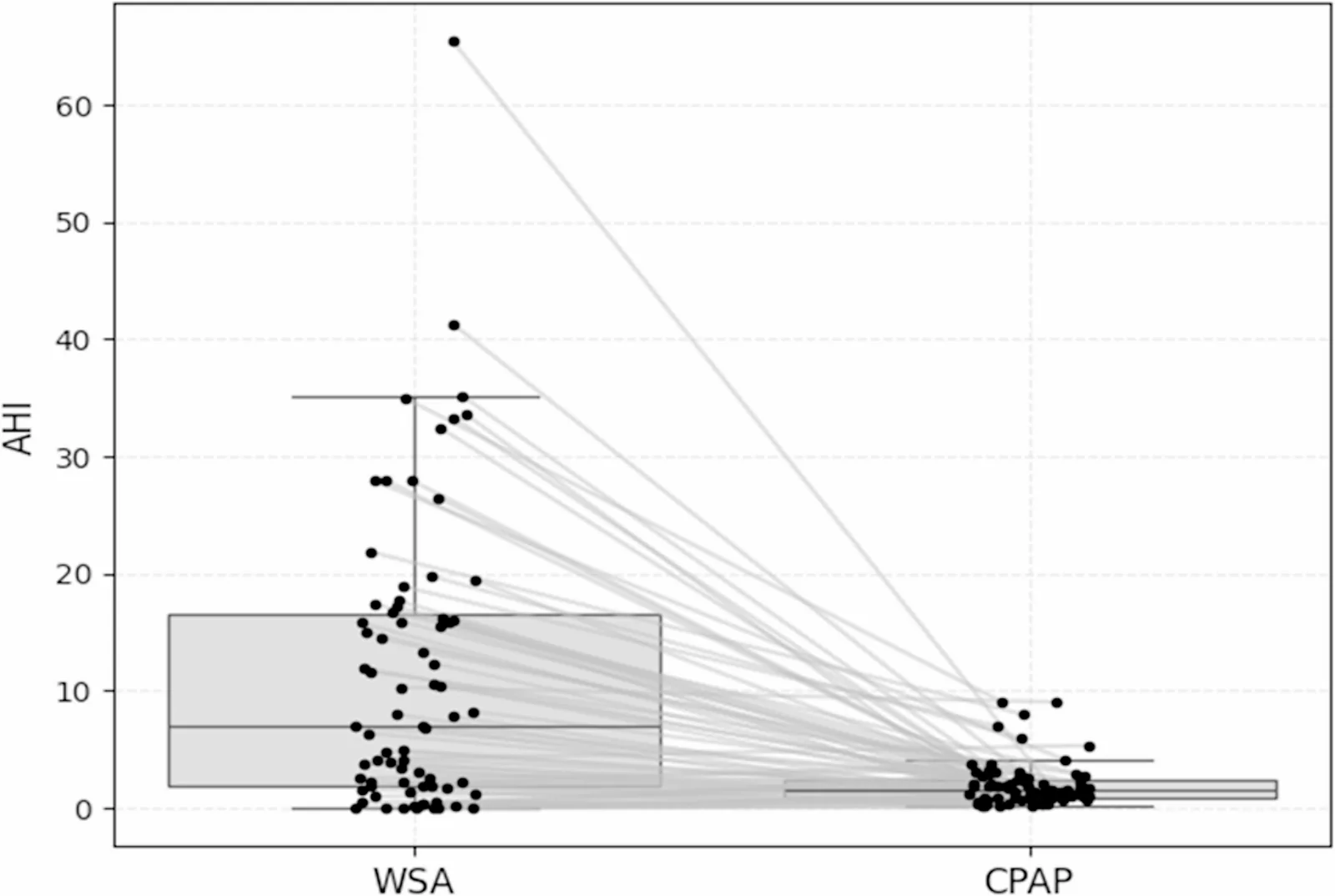
Comparison between WITHINGS Sleep AHI and CPAP residual AHI

Demographic and clinical data are summarized in Table 1 for the studied population. The WSA and the CPAP performed measurements during the same nights. However, only nights with a WSA Total Sleep Time greater than 5 h were included in the analysis, as this duration is necessary to obtain a reliable measurement of AHI. In total, 448 nights were analyzed out of 480 recorded, corresponding to an average of 6 nights per patient (Table 2). Over the 6 nights studied, the average duration of CPAP use (395.6 ± 96.6 min) was lower than WITHINGS Time in Bed (497.1 ± 69.5 min) (*p* < 0.001) and WITHINGS Total Sleeping Time (TST) (437.4 ± 62.7 min) (*p* < 0.005) (Table 2; Figs. 2 and 3). The average difference between CPAP use time and WSA TST was 41.9 ± 9.4 min. The mean CPAP AHI (2.0 ± 1.9 events/h) was significantly lower than the WSA AHI (11.3 ± 12.4 events/h) (*p* < 0.001)(Fig. 4). Table 2 shows that the intra-individual night to night AHI variation was high for the WSA (5.4 ± 5.3 events/h) whereas it was much lower for CPAP residual AHI (1.1 ± 1.5 events/h).

**Table 1:** Patients characteristics of the 75 pts included

Females	*n* (%) or Mean	STD [MIN; MAX]
	17 (22.7%)	-
Age	55.1	12.1 [23; 79]
BMI	30.8	4.7 [21.3; 24.0]
Epworth at inclusion	11.2	5.4 [1; 24]
AHI at inclusion Obst apnea Mixed apnea Central apnea Hypopnea index	44.2 15.5 2.4 1.7 24.5	20.5 [10.3–88.5] 16.0 3.0 4.6 12.6
Oxygen desaturation index (/hr)	36.6	19.7
Hypertension	30 (40%)	-
Snoring	62 (82.7%)	-
Daytime hypersomnolence	52 (69.3%)	-
Non restorative sleep	37 (49.3%)	-
Insomnia	26 (34.7%)	-
Depression	12 (16%)	-

**Table 2:** CPAP and WITHINGS Sleep Analyzer data

	*N* (%) or Average	Median	SD
Number of patients	75	-	-
Number of nights with WITHINGS data	480 (on average 6.4 nights/patient)	-	-
Number of nights with WITHINGS Sleep TST > 5 h	448 (on average 6 nights/patient)	-	-
**CPAP data**			
Duration of CPAP use (min)	389.9	403	99.2
CPAP residual AHI/hr	1.98	1.5	1.89
Duration of CPAP use (WITHINGS Sleep TST > 5 h) (min)	395.6	413.3	96.6
CPAP residual AHI (WITHINGS Sleep TST > 5 h)	2.0	1.5	1.9
Difference Sleep TST – CPAP use (min)	36.8	9.6	9.6
Difference Sleep TST – CPAP use (min) (WITHINGS Sleep TST > 5 h)	41.9	13.5	9.4
Duration of CPAP use variation intra-patient (min)	72.3	69.7	34.6
Duration of CPAP use variation intra-patient (min) (WITHINGS Sleep TST > 5 h)	69.7	62.7	35.7
CPAP AHI variation intra-patient (/hr)	1.1	1.5	1.5
CPAP AHI variation intra-patient (/hr) (WITHINGS Sleep TST > 5 h)	1.2	0.7	1.7
**WITHINGS Sleep Analyzer data**			
TIB averaged by patient (min)	486.3	476.4	74.9
TST averaged by patient (min)	426.7	419.4	70.5
TIB for WITHINGS Sleep TST > 5 h	497.1	490.1	69.5
TST for WITHINGS Sleep TST > 5 h	437.4	429.5	62.7
WITHINGS Sleep AHI (/hr) WITHINGS Sleep TST > 5 h	11.3	7.0	12.4
TIB variation intra-patient (min)	73.0	65.1	29.7
TST variation intra-patient (min)	67.9	59.7	25.8
TIB variation intra-patient (min) WITHINGS Sleep TST > 5 h	63.6	58.3	29.7
TST variation intra-patient (min) WITHINGS Sleep TST > 5 h	59.0	55.5	25.8
WITHINGS Sleep AHI variation intra-patient (/hr) WITHINGS Sleep TST > 5 h	5.4	3.7	5.3

**Fig. 5:**
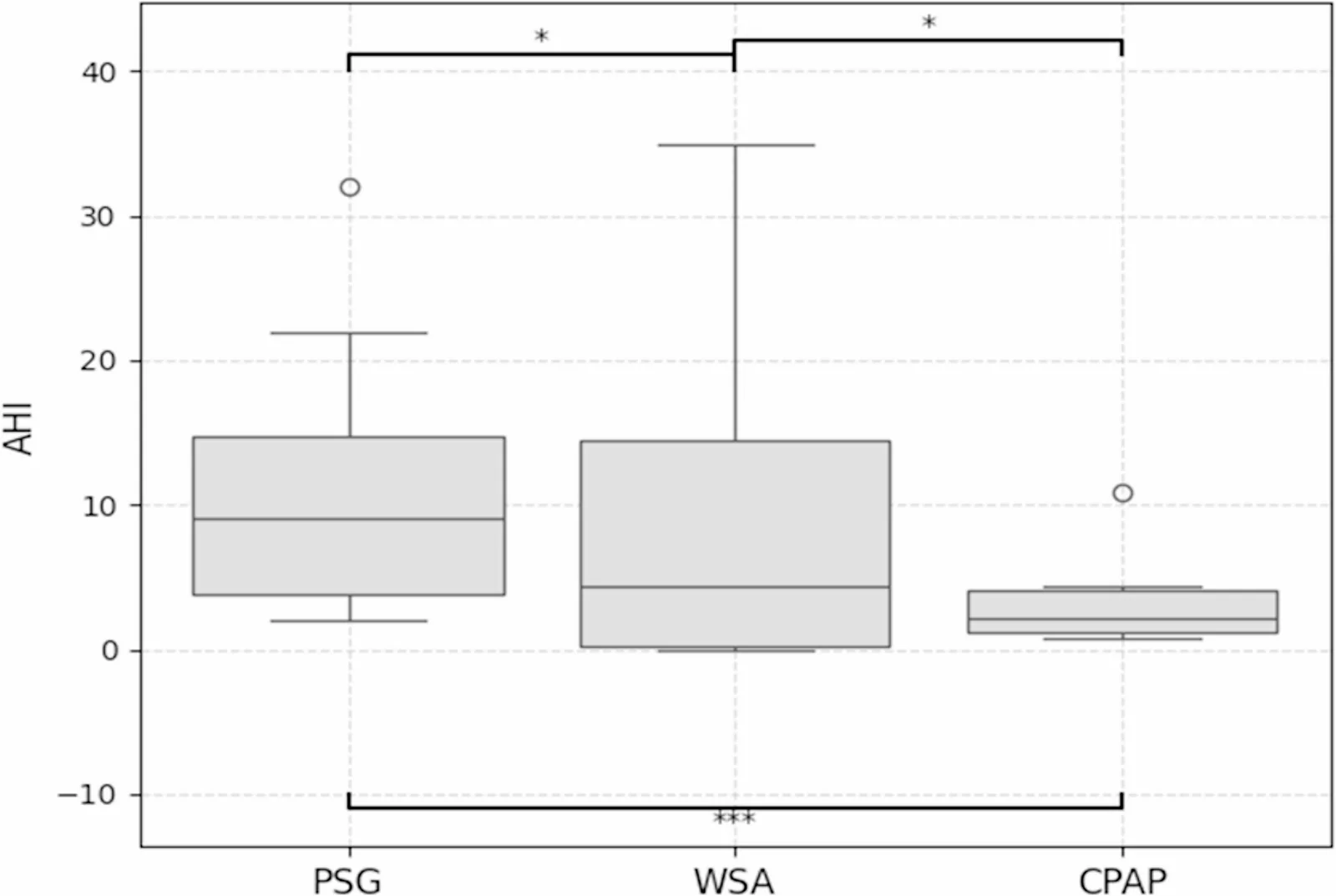
Comparison of AHI by PSG/WSA/CPAP in 14 patients

**Fig. 6:**
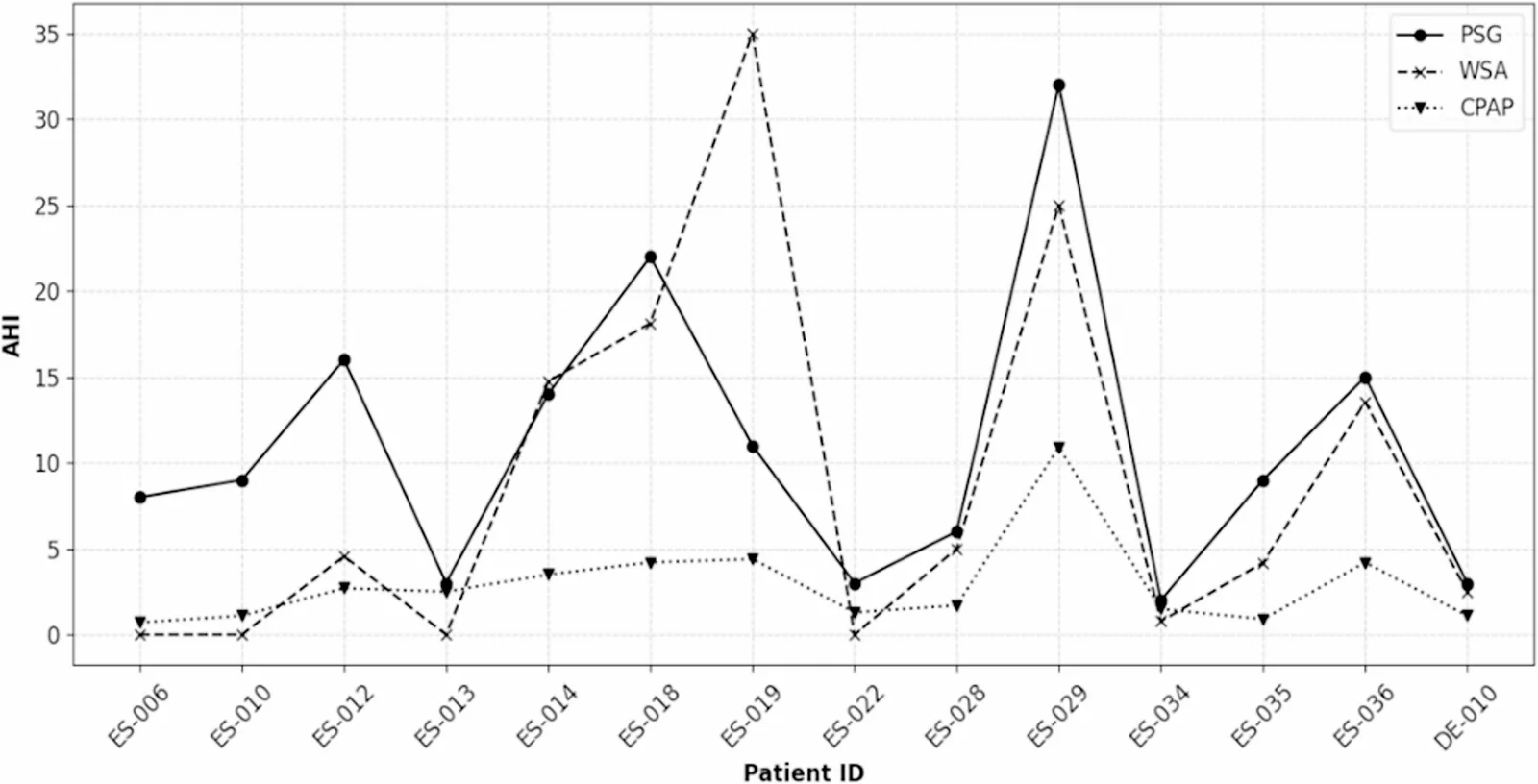
Comparison of individual AHI of the 14 patients recorded by PSG/WSA/CPAP

In the 14 patients simultaneously recorded by PSG and WSA on CPAP treatment, the CPAP use was significantly higher than the TST recorded by PSG (*p* < 0.001) which was not different from WSA TST (Table 3). AHI measured by PSG (10.9 ± 8.4 events/h) was significantly higher than WSA AHI (8.8 ± 11.0 events/h) (*p* < 0.05) which was also higher than CPAP residual AHI (2.9 ± 2.7events/h) (*p* < 0.05) (Table 3; Fig. 5). Figure 6 shows individual comparisons of AHI determined by PSG, WSA and CPAP.

**Table 3:** CPAP, WITHINGS and PSG data in 14 patients

	*N* (%) or Average	STD
Number of patients	14	-
**CPAP data**		
Duration of use (min)	483.9	83.4
CPAP AHI (/hr)	2.9	2.7
CPAP AHI “corrected” by TST (/hr)	3.8	3.9
LEAKS median (l/min)	1.1	2.4
LEAKS 95% (l/min)	12.3	10.8
**WITHINGS Sleep Analyzer data**		
TIB (min)	498.3	90.5
TST (min)	446.2	85.7
WITHINGS Sleep AHI	8.8	11.0
**PSG data**		
TST (min)	408.6	77.4
total AHI (/hr) Obstructive apneas Central apneas Hypopneas	10.9 0.5 0.5 9.9	8.4 1.1 0.7 7.4

Bland-Altman difference between PSG AHI and WSA AHI gave a bias of 2.1/h, a standard deviation of 8.3 (Figure S1). Difference between PSG AHI and CPAP AHI gave a bias of 8.0/h, a standard deviation of 6.3 (Figure S2). When CPAP residual AHI was computed over the Total Sleep Time measured by PSG (as total number of respiratory events detected by CPAP during the CPAP use time/TST), mean CPAP AHI “corrected” by TST was 3.8 ± 3.9/hr (Table 3).

## Discussion

In this study on 480 nights in 75 patients simultaneously using CPAP and WSA, a non-intrusive under-mattress sensor, TST estimated by the WSA was 41.9 min longer than CPAP use time averaged over 6 nights (*p* < 0.001). The AHI determined by the WSA was significantly higher (11.3 events/h) than residual events recorded by CPAP (2.0/h) (*p* < 0.001). In 14 of these patients simultaneously recorded at home during one night by PSG, AHI measured by PSG was significantly higher (10.9/h) compared to AHI measured by the WSA (8.8/h) and CPAP residual AHI (2.9/h).

The non-intrusive home monitoring of CPAP treatment by the WSA highlights two pitfalls in determining the effectiveness of CPAP treatment: the duration of CPAP use over the actual TST and the validity of AHI measurements by the CPAP machine. It has been suggested that CPAP compliance should be evaluated as a fraction of TST rather than an absolute value [12, 23, 24]. During the PSG, the AHI is given in relation to TST, including sleep phases and excluding wake times, whereas CPAP devices record and store the data over the total use time associated with a measurable breathing signal. Because periods of quiet wakefulness without active movements can be misclassified as sleep, actimetry-derived TST typically shows a mean difference of 40 min compared to PSG [25]. The WSA algorithm, in addition to activity, incorporates absolute and variability measures of heart rate and breathing rate to estimate TST [20]. Previous studies have shown that this approach results in an overestimation of PSG TST by 20 min in 117 normal subjects recorded at home [26] and by 26 min in 118 sleep apnea patients in the sleep laboratory [20]. Therefore, the 41.9-minute difference observed in our study between WSA TST and CPAP use time may be partially attributable to TST overestimation by the WSA. Another contributor to the observed difference is the time spent sleeping without CPAP, during which patients experience untreated OSA that is not measured by the CPAP device. In our highly adherent group of patients (average CPAP use 6h46min), the amount of sleep without CPAP was low compared to what might be expected in less adherent patients in which the WSA-CPAP discrepancy might be much higher due to the usual recurrence of apneas after discontinuation of the CPAP before the end of the night. In the 14 patients recorded by PSG, CPAP use time was longer than the actual sleep period (Table 3), explained by the study protocol: patients were instructed to wait for the technician to disconnect the equipment in the morning and therefore kept their CPAP on, while awake, until the technician arrived. A similar situation may occur in routine practice when patients keep their mask on during periods of wakefulness at night. In such cases, the CPAP residual index may be falsely low because the use time exceeds the actual sleep time as evidenced by our results of “corrected” CPAP AHI (Table 3). In a study of 300 CPAP treated patients explored by PSG, automatic detection of residual AHI by the built-in software was not reliable in a real-life context, demonstrating a high percentage of false negatives for a residual AHI > 15/h measured by PSG [27]. Statistically significant differences exist between CPAP brands when reporting residual AHI for the same patients [28]. The RESMED CPAP underestimated residual events in the whole group (Table 2) and in the subgroup with PSG where the differences between AHI estimated by WSA and by the CPAP were significant (Table 3). The RESMED algorithm does not include events during major leaks, ramp period, or central hypopneas [29]. In our study the discrepancy in AHI between the CPAP and the PSG and the mattress was not attributable to errors AHI estimation due to leaks or central apneas as indicated by the absence of significant leaks and the low central apnea index during PSG (Table 3). Therefore the underestimation of residual events is most likely due to the flow-events analysis of hypopneas, as previously observed in several CPAP devices [14, 15, 30]. Compared to AHI measured by PSG, the bias of the WSA was 2.1/h (8.3) (Figure S1) similar to the bias observed during PSG without CPAP: 1.6/h (12.5) [20]. Similarly the differences between TST measured by PSG and WSA was 37.6 min, close to the 26 min observed by Edouard et al. [20]. Therefore no interference of CPAP on WSA measurements was observed in this limited sample.

### Limitations of the study

Because the study focused on highly adherent patients to validate the WSA under CPAP, it was not possible to assess the usefulness of the WSA for estimating recurrent apneic events in non-adherent patients who interrupt CPAP use during the night. In such cases, a detailed timeline of sleep disturbances detected by the WSA, ideally synchronized with the CPAP hourly usage graph, could help identify and address these patterns.

By protocol, patients and HHCP were not allowed access to the results of the WSA, therefore we cannot evaluate the effect of WSA data on closer monitoring of treatment by the HHCP and possible empowerment by the patient. This effect could result from the demonstration to the patient of persistent breathing events when CPAP is not used over all sleeping time and also from objective information on their sleep patterns.

Any potential electromagnetic interference of the WSA on the CPAP device was impossible to directly measure at the patient’s home at night. An indirect method, measuring any difference in CPAP AHI and/or CPAP use time when the WITHINGS Sleep analyzer was installed, compared to a period without WSA, showed no significant difference (Table S1 and Figures S3 and S4).

Finally due to the PHILIPS CPAP recall, this study apply only to RESMED Airsense device and therefore these findings cannot be generalized to other CPAP platforms before a multi-device replication study. Furthermore the wide limits of agreement in the WSA vs. PSG Bland-Altman (SD 8.3/h) reflect individual variability but these findings need a more robust analysis on a larger group to draw definitive conclusions.

Sleep position and bed-partner presence were not controlled for, may also have influenced WSA signal quality and contribute to night-to-night AHI variability.

### Clinical perspectives

Although the WSA-AHI threshold and the TST–CPAP discrepancy warranting therapeutic adjustment remain undefined, the combination of residual overnight AHI, CPAP usage time, and total sleep time could enable a more accurate assessment of the actual disease alleviation achieved with CPAP under real-world home conditions on a nightly basis, compared with a single-night polysomnographic evaluation performed in the sleep laboratory after six months of treatment as proposed by Budhijara [24]. A recent study has shown that a consumer-grade oximeter wearable augmented PAP use, on average of 1.33 additional hours per night, but did not allow to determine the “total apnea burden”, including residual Apnea-Hypopnea Index (AHI) and oxygen desaturation during non-use periods as this wearable does not provide an estimation of Total Sleep Time [31]. The true alleviation, integrating both adherence over sleep time and treatment efficacy could be of value for accurate monitoring and personalized treatment, especially when evaluating long-term outcomes or making clinical adjustments. This passive longitudinal follow-up of TST and true AHI on treatment in a remote monitoring program, could facilitate the prediction of treatment responses, the optimization of titration, and the enhancement of adherence [32].

The evaluation of sleep patterns during CPAP could also allow an easier detection of associated comorbid insomnia. The frequent clinical phenotype COMISA remains insufficiently recognized in patients at high risk of poor adherence and intolerance to CPAP while having a higher prevalence and incidence of cardiovascular diseases compared with OSA alone [33]. This population requires personalized care including cognitive behavioral therapy for insomnia which could also possibly be monitored by the WSA. Finally it remains to be demonstrated if this non-intrusive monitoring could be of value in the titration and follow-up of other OSA treatment modalities such as mandibular advancement devices and hypoglossal electrical stimulation.

## Conclusion

Non-intrusive home monitoring of AHI and TST, using the WSA, an under-mattress sensor, allowed to estimate on several nights, TST which was higher than CPAP use in a highly adherent group of 75 patients. Residual events reported by RESMED Airsense CPAP were underestimated compared to WSA whose AHI was closer to PSG AHI. This device could be of value particularly in poorly adherent patients to increase empowerment and for accurate monitoring of personalized treatments.

## Supplementary Information

Below is the link to the electronic supplementary material.

Supplementary Material 1 (DOCX 87.7 KB)

## Data Availability

Deidentified data that support the findings of this study, including individual data, are available from the corresponding author upon request subject to ethical and data custodian (WITHINGS) approval.

## References

[1] Benjafield AV, Ayas NT, Eastwood PR, Heinzer R, Ip MSM, Morrell MJ, Nunez CM et al (2019) Estimation of the global prevalence and burden of obstructive sleep apnoea: a literature-based analysis. Lancet Respir Med 7:687–698. 10.1016/S2213-2600(19)30198-5 31300334 10.1016/S2213-2600(19)30198-5 PMC7007763

[2] Heinzer R, Vat S, Marques-Vidal P, Marti-Soler H, Andries D, Tobback N, Mooser V et al (2015) Prevalence of sleep-disordered breathing in the general population: the HypnoLaus study. Lancet Respir Med 3:310–318. 10.1016/S2213-2600(15)00043-0 25682233 10.1016/S2213-2600(15)00043-0 PMC4404207

[3] Patil SP, Billings ME, Bourjeily G, Collop NA, Gottlieb DJ, Johnson KG, Kimoff RJ, Pack AI (2024) Long-term health outcomes for patients with obstructive sleep apnea: placing the Agency for Healthcare Research and Quality report in context—a multisociety commentary. J Clin Sleep Med 20:135–149. 10.5664/jcsm.10832 37904571 10.5664/jcsm.10832 PMC10758567

[4] Giles T, Lasserson T, Smith B, White J, Wright J, Cates C (2006) Continuous positive airways pressure for obstructive sleep apnoea in adults. *Cochrane Database of Systematic Reviews*, ed. The Cochrane Collaboration, CD001106.pub2. John Wiley & Sons, Ltd, Chichester, UK. 10.1002/14651858.CD001106.pub2. 10.1002/14651858.CD001106.pub2 16437429

[5] Sánchez-de-la-Torre M, Gracia-Lavedan E, Benitez ID, Sánchez-de-la-Torre A, Moncusí-Moix A, Torres G, Loffler K et al (2023) Adherence to CPAP treatment and the risk of recurrent cardiovascular events: a meta-analysis. JAMA 330:1255–1265. 10.1001/jama.2023.17465 37787793 10.1001/jama.2023.17465 PMC10548300

[6] Sabil A, Launois C, Trzepizur W, Goupil F, Pigeanne T, Launois S, Leclair-Visonneau L et al (2024) Association of positive airway pressure termination with mortality and non-fatal cardiovascular events in patients with obstructive sleep apnoea. Thorax 79:1077. 10.1136/thorax-2024-221689 39095088 10.1136/thorax-2024-221689

[7] Baratta F, Pastori D, Bucci T, Fabiani M, Fabiani V, Brunori M, Loffredo L et al (2018) Long-term prediction of adherence to continuous positive air pressure therapy for the treatment of moderate/severe obstructive sleep apnea syndrome. Sleep Med 43:66–70. 10.1016/j.sleep.2017.09.032 29482815 10.1016/j.sleep.2017.09.032

[8] Pépin J-L, Bailly S, Rinder P, Adler D, Szeftel D, Malhotra A, Cistulli P et al (2021) CPAP therapy termination rates by OSA phenotype: a French nationwide database analysis. J Clin Med 10:936. 10.3390/jcm10050936 33804319 10.3390/jcm10050936 PMC7957656

[9] Patil SP, Ayappa IA, Caples SM, Kimoff RJ, Patel SR, Harrod CG (2019) Treatment of adult obstructive sleep apnea with positive airway pressure: an American Academy of Sleep Medicine clinical practice guideline. J Clin Sleep Med 15:335–343. 10.5664/jcsm.7640 30736887 10.5664/jcsm.7640 PMC6374094

[10] Labarca G, Schmidt A, Dreyse J, Jorquera J, Barbe F (2021) Telemedicine interventions for CPAP adherence in obstructive sleep apnea patients: systematic review and meta-analysis. Sleep Med Rev 60:101543. 10.1016/j.smrv.2021.101543 34537668 10.1016/j.smrv.2021.101543

[11] Mokhlesi B, Finn LA, Hagen EW, Young T, Hla KM, Van Cauter E, Peppard PE (2014) Obstructive sleep apnea during REM sleep and hypertension. Results of the Wisconsin sleep cohort. Am J Respir Crit Care Med 190:1158–1167. 10.1164/rccm.201406-1136OC 25295854 10.1164/rccm.201406-1136OC PMC4299639

[12] Grote L, Hedner J, Grunstein R, Kraiczi H (2000) Therapy with nCPAP: incomplete elimination of sleep related breathing disorder. Eur Respir J 16:921–927. 10.1183/09031936.00.16592100 11153593 10.1183/09031936.00.16592100

[13] Richter M, Schroeder M, Domanski U, Schwaibold M, Nilius G (2023) Reliability of respiratory event detection with continuous positive airway pressure in moderate to severe obstructive sleep apnea — comparison of polysomnography with a device-based analysis. Sleep Breath 27:1639–1650. 10.1007/s11325-022-02740-w 36394692 10.1007/s11325-022-02740-w PMC9669528

[14] Richter M, Schroeder M, Nilius G (2024) Accuracy of respiratory event indices downloaded from positive airway pressure devices: can they be relied upon when making treatment decisions? Curr Opin Pulm Med. 10.1097/MCP.0000000000001113 39115392 10.1097/MCP.0000000000001113

[15] Reiter J, Zleik B, Bazalakova M, Mehta P, Thomas RJ (2016) Residual events during use of CPAP: prevalence, predictors, and detection accuracy. J Clin Sleep Med 12:1153–1158. 10.5664/jcsm.6056 27166303 10.5664/jcsm.6056 PMC4957193

[16] Ni Y-N, Thomas RJ (2022) A longitudinal study of the accuracy of positive airway pressure therapy machine-detected apnea-hypopnea events. J Clin Sleep Med 18:1121–1134. 10.5664/jcsm.9814 34886948 10.5664/jcsm.9814 PMC8974380

[17] Sharafkhaneh A, Benjafield AV, Penzel T (2026) Improving PAP adherence through consumer wearables: a promising step forward. SLEEP zsag007. 10.1093/sleep/zsag007 41530893 10.1093/sleep/zsag007

[18] Kapur Vishesh K, Auckley DH, Chowdhuri S, Kuhlmann DC, Mehra R, Ramar K, Harrod CG (2017) Clinical practice guideline for diagnostic testing for adult obstructive sleep apnea: an American Academy of Sleep Medicine clinical practice guideline. J Clin Sleep Med 13:479–504. 10.5664/jcsm.6506 28162150 10.5664/jcsm.6506 PMC5337595

[19] Yang R-Y, Bendjoudi A, Buard N, Boutouyrie P (2019) Pneumatic sensor for cardiorespiratory monitoring during sleep. Biomed Phys Eng Express 5:055014. 10.1088/2057-1976/ab3ac9

[20] Edouard P, Campo D, Bartet P, Yang R-Y, Bruyneel M, Roisman G, Escourrou P (2021) Validation of the Withings Sleep Analyzer, an under-the-mattress device for the detection of moderate-severe sleep apnea syndrome. J Clin Sleep Med 17:1217–1227. 10.5664/jcsm.9168 33590821 10.5664/jcsm.9168 PMC8314651

[21] Lechat B, Naik G, Reynolds A, Aishah A, Scott H, Loffler KA, Vakulin A et al (2022) Multinight prevalence, variability, and diagnostic misclassification of obstructive sleep apnea. Am J Respir Crit Care Med 205:563–569. 10.1164/rccm.202107-1761OC 34904935 10.1164/rccm.202107-1761OC PMC8906484

[22] Pinilla L, Ching Li C-C, Eckert DJ (2025) Diagnostic modalities in sleep disordered breathing: current and emerging technology and its potential to transform diagnostics. Respirology 30:286–302. 10.1111/resp.70012 40032579 10.1111/resp.70012 PMC11965016

[23] Pfammatter AF, Hughes BO, Tucker B, Whitmore H, Spring B, Tasali E (2022) The development of a novel mHealth tool for obstructive sleep apnea: tracking continuous positive airway pressure adherence as a percentage of time in bed. J Med Internet Res 24:e39489. 10.2196/39489 36469406 10.2196/39489 PMC9764150

[24] Budhiraja R, Batool-Anwar S, Quan SF (2025) Mean disease alleviation as a comprehensive metric for evaluating continuous positive airway pressure therapy in obstructive sleep apnea: establishing reference values and clinical significance. J Clin Sleep Med 21:973–982. 10.5664/jcsm.11592 39916590 10.5664/jcsm.11592 PMC12134571

[25] Smith MT, McCrae CS, Cheung J, Martin JL, Harrod CG, Heald JL, Carden KA (2018) Use of actigraphy for the evaluation of sleep disorders and circadian rhythm sleep-wake disorders: an American Academy of Sleep Medicine systematic review, meta-analysis, and GRADE assessment. J Clin Sleep Med 14:1209–1230. 10.5664/jcsm.7228 29991438 10.5664/jcsm.7228 PMC6040804

[26] Stefanos M-A, De Laboulaye G, Campo D, De Gourcuff M, Escourrou P, Matrot B, Islind AS, Geoffroy PA (2025) Evaluation of a contactless sleep monitoring device for sleep stage detection against home polysomnography in a healthy population. Health Informatics J. 10.1101/2025.05.06.25326860 10.2196/77033 PMC13046097 41926681

[27] Fanfulla F, D’Artavilla Lupo N, Malovini A, Arcovio S, Prpa A, Mogavero MP, Pronzato C, Bonsignore MR (2021) Reliability of automatic detection of AHI during positive airway pressure treatment in obstructive sleep apnea patients: a “real-life study.” Respir Med 177:106303. 10.1016/j.rmed.2021.106303 33444877 10.1016/j.rmed.2021.106303

[28] Midelet A, Borel J-C, Tamisier R, Le Hy R, Schaeffer M-C, Daabek N, Pépin J-L, Bailly S (2021) Apnea-hypopnea index supplied by CPAP devices: time for standardization? Sleep Med 81:120–122. 10.1016/j.sleep.2021.02.019 33667996 10.1016/j.sleep.2021.02.019

[29] Vidal C, Mallet J-P, Skinner S, Gilson R, Gaubert O, Prigent A, Gagnadoux F, Bourdin A, Molinari N, Jaffuel D (2025) Concerns arising from the calculation of the apnea-hypopnea index during CPAP-telemonitoring of patients with obstructive sleep apnea. Respir Res 26:244. 10.1186/s12931-025-03324-4 40652228 10.1186/s12931-025-03324-4 PMC12255124

[30] Iftikhar IH, BaHammam A, Jahrami H, Ioachimescu O (2023) Accuracy of residual respiratory event detection by CPAPs: a meta-analysis. Sleep Breath 27:1759–1768. 10.1007/s11325-023-02780-w 36715836 10.1007/s11325-023-02780-w

[31] Fung CH, Mak S, Ash G, Der-McLeod E, Ereso BSJ, Naeem S, Liu J et al (2025) Augmenting management of obstructive sleep apnea with consumer wearable devices to increase use of positive airway pressure therapy: a pilot randomized trial. Sleep zsaf342. 10.1093/sleep/zsaf342 10.1093/sleep/zsaf342 PMC13475902 41159689

[32] Timmis JK, Schorr KA, Yüksel R, Van Den Broek T, Overeem S, Smid DJ, Van Den Brink WJ, Haring NL (2026) Toward patient-centric digital monitoring of obstructive sleep apnea: mixed methods study. J Med Internet Res 28:e82460. 10.2196/82460 41505749 10.2196/82460 PMC12828318

[33] Sweetman A, Osman A, Lack L, Crawford M, Wallace D (2023) Co-morbid insomnia and sleep apnea (COMISA): recent research and future directions. Curr Opin Pulm Med. 10.1097/MCP.0000000000001007 37642477 10.1097/MCP.0000000000001007

