# Supplementary material for: Comparison of under-mattress sensor and CPAP data for home monitoring in obstructive sleep apnea
Source: Sleep Breath. 2026 Jun 22;30(4):194. doi: 10.1007/s11325-026-03741-9 (PMC13287269; doi:10.1007/s11325-026-03741-9)
Supplement: Supplementary file 1 — Supplementary Material 1 (DOCX 87.7 KB) [file 11325_2026_3741_MOESM1_ESM.docx]

**Comparison of Under-Mattress Sensor and CPAP Data for Home Monitoring in Obstructive Sleep Apnea R-Y Yang^1^, et al**

Supplemental data :

|  | **N (%) or Average** | **STD** |
| --- | --- | --- |
| Number of patients | 20 | - |
| CPAP data without WITHINGS Sleep installed | | |
| Number of nights | 76 (in average 3.8 nights/patient) | - |
| Duration of use averaged by patient (min) | 410.3 | 52.2 |
| CPAP AHI averaged by patient | 1.9 | 2.6 |
| CPAP data with WITHINGS Sleep installed | | |
| Number of nights | 130 (in average 6.5 nights/patient) | - |
| Duration of use averaged by patient (min) | 420.6 | 68.9 |
| CPAP AHI averaged by patient | 1.5 | 1.2 |

Table S 1: CPAP data for nights without and WITHINGS Sleep Analyzer


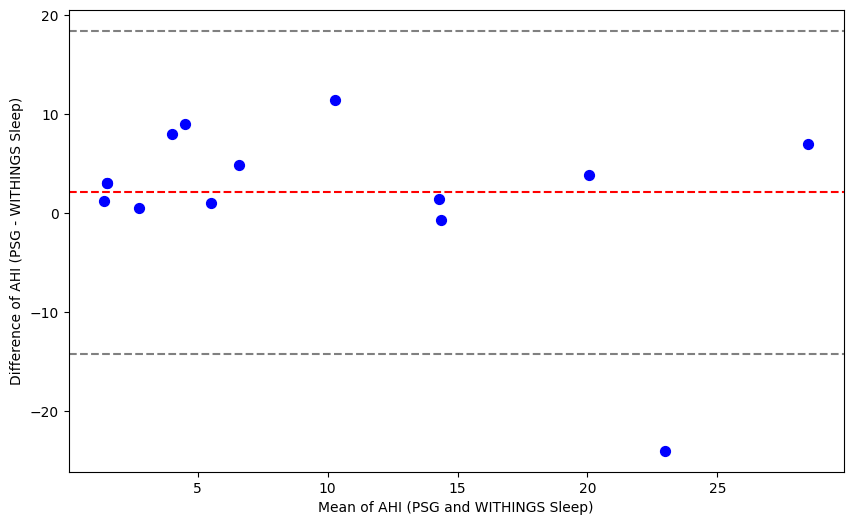


###### Fig. S1 : Bland-Altman between PSG AHI and WITHINGS Sleep Analyzer AHI.

Difference between PSG AHI and WITHINGS Sleep AHI gave a bias of 2.1, a standard deviation of 8.3 and a mean absolute error (MAE) of 5.6


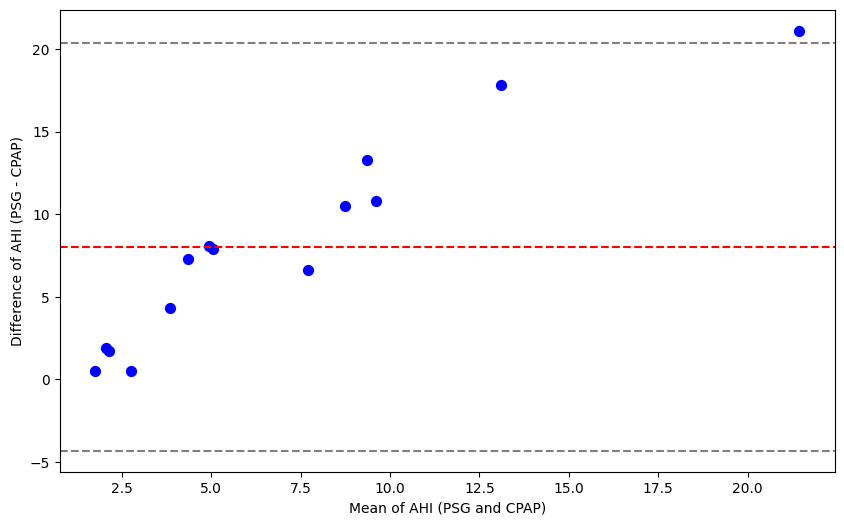


###### Fig. S2 : Bland-Altman between PSG AHI and CPAP AHI.

Difference between PSG AHI and CPAP AHI gave a bias of 8.0, a standard deviation of 6.3 and a mean absolute error (MAE) of 8.0


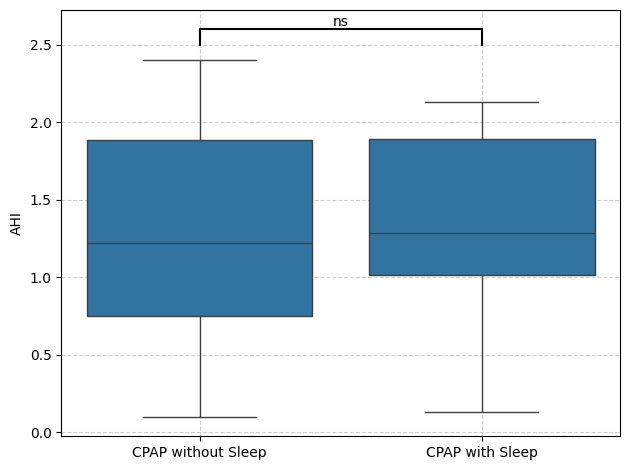


###### Fig. S 3 : Boxplot of CPAP AHI for nights without and with WITHINGS Sleep Analyzer installed (Wilcoxon test, ns: p-value ≥ 0.05)


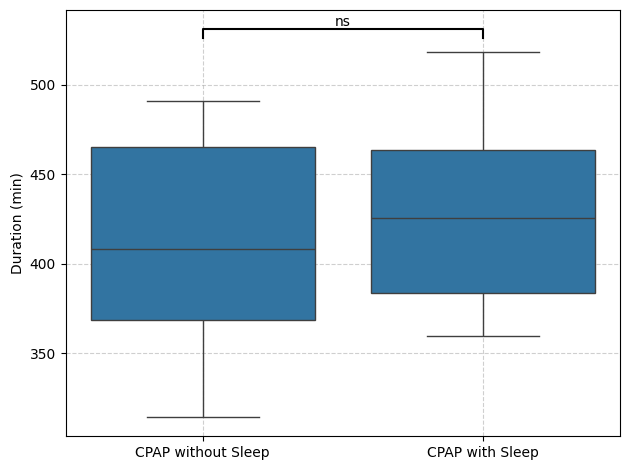


###### Fig. S 4 : Boxplot of CPAP duration of use for nights without and with WITHINGS Sleep Analyzer installed (Wilcoxon test, ns: p-value ≥ 0.05)
